# Comprehensive pregnancy dental benefits improved dental coverage and increased dental care utilization among Medicaid-enrolled pregnant women in Virginia

**DOI:** 10.3389/froh.2022.989659

**Published:** 2022-09-20

**Authors:** Shillpa Naavaal, David W. Harless

**Affiliations:** ^1^ Department of Dental Public Health and Policy, School of Dentistry, Virginia Commonwealth University, Richmond, VA, United States; ^2^ Oral Health Core, Institute for Inclusion, Inquiry, and Innovation, Virginia Commonwealth University, Richmond, VA, United States; ^3^ Department of Economics, School of Business, Virginia Commonwealth University, Richmond, VA, United States

**Keywords:** dental insurance, dental care utilization, pregnant women, Medicaid policy analysis, Medicaid dental program

## Abstract

**Objectives:**

To evaluate the changes in dental insurance and utilization among pregnant women before and after the pregnancy Medicaid dental benefit policy implementation in 2015 in Virginia.

**Methods:**

We used pooled cross-sectional data from six cycles of the Virginia Pregnancy Risk Assessment Monitoring System on women aged ≥21 years. Using logistic regression models and a difference-in-difference design, we compared the effects of policy implementation on dental insurance and utilization between pre-policy (2013–2014) and post-policy period (2016–2019) among women enrolled in Medicaid (treatment, *N* = 1,105) vs. those with private insurance (control, *N* = 2,575). A *p*-value of 0.05 was considered significant.

**Results:**

Among Medicaid-enrolled women, the report of dental insurance (71.6%) and utilization (37.7%) was higher in the post-period compared to their pre-period (44.4% and 30.3%, respectively) estimates but still remained lower than the post-period estimates among women with private insurance (88.0% and 59.9%, respectively). Adjusted analyses found that Medicaid-enrolled women had a significantly greater change in the probability of reporting dental insurance in all post-period years than women with private insurance, while the change in the probability of utilization only became statistically significant in 2019. In 2019, there was a 16 percentage point increase (95% CI = 0.05, 0.28) in the report of dental insurance and a 17 percentage point increase (95% CI = 0.01–0.33) in utilization in treatment group compared to controls.

**Conclusions:**

The 2015 pregnancy Medicaid dental benefit increased dental insurance and dental care utilization among Medicaid-enrolled women and reduced associated disparities between Medicaid and privately insured groups.

## Introduction

Good oral health during pregnancy is an essential part of maternal and child's overall health (1). Oral health problems such as periodontal disease during pregnancy is associated with an increased risk of adverse pregnancy and fetal health outcomes, including preterm birth and low birth weight (2, 3). Recognizing the importance of oral health for pregnant women, various state and national programs and professional organizations have developed educational materials, guidelines, and recommendations to include oral health in routine prenatal care (1, 4, 5). However, despite clear support and guidance, only 46% of pregnant women visited a dentist during pregnancy in 2017 (6).

Multiple factors can play a role in influencing oral health access and the use of dental services among pregnant women (7, 8). Several studies have reported that women in racial/ethnic minority groups, those with low income, or those with low education face more barriers to accessing high-quality and affordable dental care during pregnancy and postpartum and have lower dental care utilization (9–11). An important system-level barrier to oral health use is the lack of dental insurance. A study using the Virginia Pregnancy Risk Assessment Monitoring System (PRAMS) data from 2012 to 2014 found that women with dental insurance had 3.5 times higher odds of a dental visit than women who did not have dental insurance (12). A recent study found low levels of oral health knowledge and limited awareness of available dental benefits among reproductive-age women enrolled in Medicaid (13).

Medicaid serves as a primary source of health care coverage for a majority of low-income pregnant women. Federal regulations require “pregnancy-related service” coverage under Medicaid, but oral health remains optional. As of 2019, 22 states provided comprehensive dental coverage for Medicaid-enrolled pregnant women, 19 provided limited dental coverage, seven provided only emergency dental care coverage, and two states offered no dental coverage (14).

In 2015, Virginia added a comprehensive dental coverage benefit for Medicaid-enrolled women age 21 years and older during pregnancy and 60 days postpartum. This dental benefit was implemented after state oral health surveillance and needs assessment data indicated a substantial unmet need for oral health care for pregnant women and infants. The governor issued emergency regulations to provide comprehensive dental coverage to pregnant women enrolled in Medicaid and Family Access to Medical Insurance Security (referred to as Medicaid from here on) programs commencing in March 2015 [17]. Before 2015, emergency extractions were the only dental service coverage available to pregnant Medicaid enrollees in Virginia.

To date, there has been no evaluation of the impact of this comprehensive dental benefit and PRAMS data provides this opportunity. Furthermore, dental care utilization depends not only on the existence but also on the enrollee's awareness of the dental benefit. We hypothesized that there would be an increase in the report of dental insurance and utilization in the post-policy years among Medicaid-enrolled pregnant women compared to women with private health insurance. Thus, the study objective was to evaluate the impact of the comprehensive dental benefit on self-reported dental insurance coverage and dental care utilization among Medicaid-enrolled pregnant women in Virginia using a quasi-experimental difference-in-difference design.

## Methods

### Data source and study population

This quasi-experimental study included the Virginia PRAMS data from 2013 to 2014 (pre-policy period) and 2016–2019 (post-policy period). The Virginia Department of Health, in collaboration with the Centers for Disease Control and Prevention (CDC), collects Virginia PRAMS data annually. Every year, mothers who gave live birth are randomly chosen from the Virginia birth certificate registry to participate in the PRAMS survey. The data is collected *via* mail/telephone survey and captures women's experiences before, during, and just after pregnancy. Further details on the PRAMS methodology are available elsewhere (15). The Virginia PRAMS data from Phase 7 (2013–2014) and Phase 8 (2016–2019) surveys were obtained from the Virginia Department of Health. This study was reviewed and approved by the Institutional Review Board at the Virginia Commonwealth University and the Virginia Department of Health. Data from 2015 was excluded as the new dental benefit was only in effect for part of the year. Inclusion criteria included women age 21 and above and those with Medicaid or private health insurance during pregnancy. Women under 21 years of age were excluded because the Medicaid pregnancy dental benefit applied only to women aged 21 and above.

The unweighted sample size varied by year; 2013 and 2014 had fewer than 400 respondents each, whereas the number in the post-period years ranged from 469 in 2016 to 858 in 2018 (2017 had 842 respondents, and 2019 had 743 respondents). Among the 4,027 unweighted respondents in 2013–2014 and 2016–2019 who reported having either Medicaid or private health insurance, 97 (2.4%) did not answer one or both of the questions on dental insurance and utilization and thus were excluded. We also excluded the 183 (4.5%) respondents who did not report their age or who were less than 21 years old, as well as 64 (1.6%) other respondents who had missing information for other control variables. The final analytic sample included 3,680 women, 1,105 with Medicare coverage and 2,575 with private insurance, reflecting a total population of 402,884 pregnant women in Virginia who gave live birth during the study period.

### Study variables

The first outcome was “self-reported dental insurance status,” reflecting responses when women were asked if they had insurance that covered dental care during pregnancy. The second outcome was “dental care utilization,” reflecting responses when women were asked whether they had their teeth cleaned by a dentist or dental hygienist during their most recent pregnancy. The variable dental care utilization only estimates dental cleaning and does not capture all dental care services that could have been received (e.g., treatment services and other non-preventive dental care).

The primary exposure of interest was the implementation of a comprehensive dental coverage benefit policy in 2015 for Medicaid-enrolled pregnant women. To evaluate the impact of this new benefit, we compared the outcomes for Medicaid enrollees (treatment group) during the pre- (2013–2014) and post-periods (2016–2019) to the outcomes for women with private health insurance (control group) from any source (work, parent, company, or exchange). Private and Medicaid health insurance status was defined based on the participant's response to the following question, “During your most recent pregnancy, what kind of health insurance did you have for your prenatal care?” The following sociodemographic control variables were also included based on prior research and literature review (12, 16, 17): maternal age (21–25 years, 26–30 years, 31–35 years, and >=36 years); race (White, Black, or other); ethnicity (Hispanic or non-Hispanic), education (less than high school, high school graduate, some college, and a bachelor's degree or higher); marital status (married or not), and location (urban or rural).

### Statistical analyses

All estimates incorporate Virginia PRAMS survey weights. A *p*-value less than 0.05 in a Wald t-test was considered significant.

Data from the six cycles of the Virginia PRAMS were pooled. Study population characteristics were examined for the private insurance and Medicaid groups during the pre-and post-periods, and the difference between the pre-and post-period estimates was calculated for each group and tested for significance. We then examined the distribution of dental insurance and the distribution of dental utilization overall and stratified by the presence of dental insurance across included years for Medicaid and private insurance groups.

Since both self-reported dental insurance and utilization outcomes were binary, we used multivariable logistic regression. A difference-in-difference design was used to estimate the effect of the 2015 Medicaid dental benefit on the study outcomes for the Medicaid group. This approach relies on the assumption that in the absence of pregnancy dental benefit, the change in the outcomes for Medicaid enrollees (treatment group) would have been the same as the change in outcomes for women with private insurance (control group). We describe our model results using average marginal effects, which estimate the change in the probability of an outcome for an indicated group compared to a reference group. The effect of the dental policy is estimated using Medicaid-year interaction variables for each post-period year; average marginal effects for these variables estimate the average treatment effect on the treated (18, 19). All analyses were conducted using Stata version 16 and appropriately accounted for the complex sampling design of PRAMS using survey weights and design variables representing the Virginia population. Each model included fixed effects for years and was controlled for age, race, ethnicity, education, location, and marital status.

## Results

Table 1 shows the distribution of sample characteristics by health insurance status in the pre- and post-periods. In the overall study population, 67.7% of women had private health insurance, and 32.3% were enrolled in Medicaid. In the private insurance group, approximately 88.0% of women reported having dental insurance in both the pre-and post-periods, while in the Medicaid group, the percentage reporting dental insurance increased significantly from 44.4% to 71.6% in the post-period. In the private insurance group, 56.8% of women utilized dental care in the pre-period and 59.9% in the post-period. Among Medicaid enrollees, the utilization percentage was higher in the post-period (37.7%) compared to the pre-period (30.3%), but both percentages were much lower compared to the private insurance group. The significance tests examined the difference in percentages between the pre-and post-periods among the two groups. The 27.2 percentage point increase in reporting dental insurance among Medicaid enrollees was highly significant, but while the absolute change in dental care utilization percentage was twice as large in the Medicaid group (7.4 percentage points) compared to the private insurance group (3.1 points), neither difference was statistically significant. There were no statistically significant differences in included sociodemographic characteristics in the Medicaid group during the two periods, but in the private insurance group, there was a significant increase in Hispanics and a significant decrease in the 26–30 age group in the post-period.

**Table 1:** Changes in outcomes and sociodemographic characteristics among private and Medicaid enrolled women between pre- (2013–2014) and post-period (2016–2019) in Virginia.

	Private (67.7%)			Medicaid (32.3%)		
	Pre %	Post %	Difference	Pre %	Post %	Difference
Dental Insurance						
Yes	87.7	88.0	0.3	44.4	71.6	27.2^c^
No	12.3	12.0		55.6	28.4	
Teeth Cleaned						
Yes	56.8	59.9	3.1	30.3	37.7	7.4
No	43.2	40.1		69.7	62.3	
Age in years						
21–25 (Ref.)	8.0	10.2		39.0	39.0	
26–30	37.6	29.9	−7.7^b^	30.6	33.8	3.2
31–35	35.2	39.3	4.1	22.2	18.2	−4.0
36 and above	19.2	20.6	1.4	8.1	9.0	0.8
Race						
White (Ref.)	77.8	73.1		57.4	48.2	
Black	9.3	11.1	1.7	28.6	37.6	9.1
Asian/other	12.9	15.8	3.0	14.0	14.2	0.2
Ethnicity						
Non-Hispanic (Ref.)	96.1	92.9		80.8	86.9	
Hispanic	3.9	7.1	3.2^a^	19.2	13.1	−6.1
Education						
Less than High School	^d^	1.5	−0.1	11.9	10.6	−1.3
High School Graduate	16.5	13.9	−2.7	38.7	47.4	8.6
Some College	18.4	21.6	3.2	32.0	33.5	1.5
Bachelors or higher (Ref.)	63.5	63.1		17.4	8.5	
Location						
Urban (Ref.)	90.8	94.2		76.9	84.4	
Rural	9.2	5.8	−3.5	23.1	15.6	−7.5
Marital Status						
Married (Ref.)	88.4	87.4		34.9	31.0	
Not Married	11.6	12.6	0.9	65.1	69.0	4.0

^a^ difference in post percentage minus pre percentage significant at the 0.05 level.

^b^ difference in post percentage minus pre percentage significant at the 0.01 level.

^c^ difference in post percentage minus pre percentage significant at the 0.001 level.

^d^ estimate is suppressed because <10 unweighted observations.

**Figure 1:**
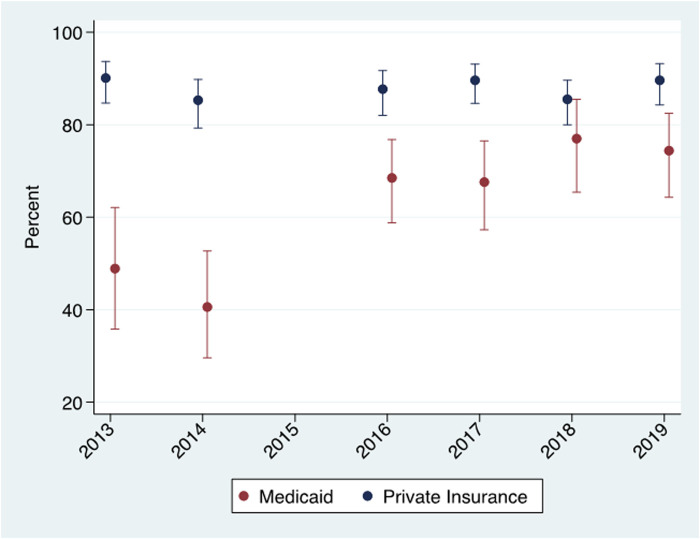
Dental insurance* report by year among Medicaid enrolled and privately insured women. Virginia PRAMS 2013–2014 and 2016–2019 data. *Dental insurance measured during pregnancy.

Figure 1 shows the estimates and 95% confidence intervals for percent reporting dental insurance by type of health insurance and year. In the private insurance group, the percent reporting dental insurance did not change significantly and hovered between 85% (2014) and 90% (2013 and 2019). There was, however, a significant increase in the percent reporting dental insurance among Medicaid-enrolled women from 40.6% in 2014 to 68.5% in 2016 and increasing to 74.4% in 2019. All Medicaid-enrolled women had comprehensive dental coverage in the post-period, yet in 2019—four years after the new dental benefit became operative—25.6% still reported having no dental insurance.

**Figure 2:**
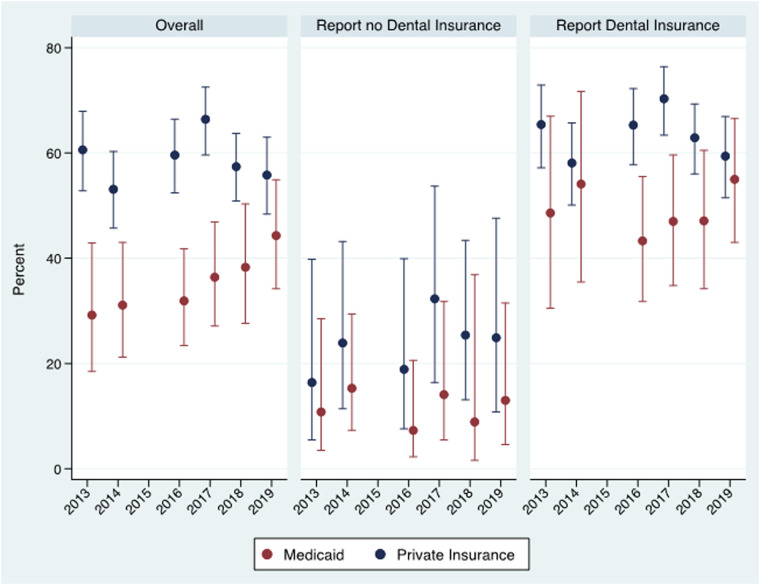
Dental care utilization by year overall and by self-reported dental insurance* status among Medicaid enrolled and privately insured women. Virginia PRAMS 2013–2014 and 2016–2019 data. *Dental insurance measured during pregnancy.

Figure 2 provides estimates and 95% confidence intervals for the percent reporting dental care utilization overall and stratified by dental insurance status. Overall, dental care utilization saw an increasing trend in the post-period years among Medicaid-enrolled women, but it remained lower than women with private insurance. In 2019, 44.3% of Medicaid-enrolled women utilized dental care compared to 55.8% in the private insurance group. The dental care utilization estimates stayed uniformly lower for Medicaid enrollees even after conditioning on self-reported dental insurance status. Among women who reported no dental insurance, dental care utilization did not show any significant trend over the study years. It ranged from 16.4% in 2013 to 32.3% in 2017 in the private insurance group and from 7.3% in 2016 to 15.3% in 2014 in the Medicaid group.

Among women who reported they had dental insurance, the utilization estimates for the private health insurance group varied between a low of 58.1% in 2014 and a high of 70.3% in 2017, with no consistent trend over the study years. Among Medicaid enrollees reporting they had dental insurance in the pre-period, 48.6% reported utilization in 2013 and 54.1% in 2014. In 2016, when all Medicaid enrollees had dental insurance, only 43.3% reported utilization, and the percentage increased slightly in 2017 and increased further to 55% in 2019.

The difference-in-difference design relies on the “parallel trends” assumption. This assumption postulates that the change in outcome variables would have been the same for the treatment (Medicaid) and the control (private insurance) group in the absence of the pregnancy dental benefit. Table 1 and Figures 1, 2 are generally consistent with this assumption. The percentage of women reporting dental insurance declined for both groups between 2013 and 2014 (Figure 1), while dental care utilization increased slightly for both groups among women reporting no dental insurance (Figure 2). The only evidence inconsistent with the parallel trends assumption was the opposite change in utilization between 2013 and 2014 among those reporting dental insurance, but the change in utilization between the years was not statistically significant for either group.

**Figure 3:**
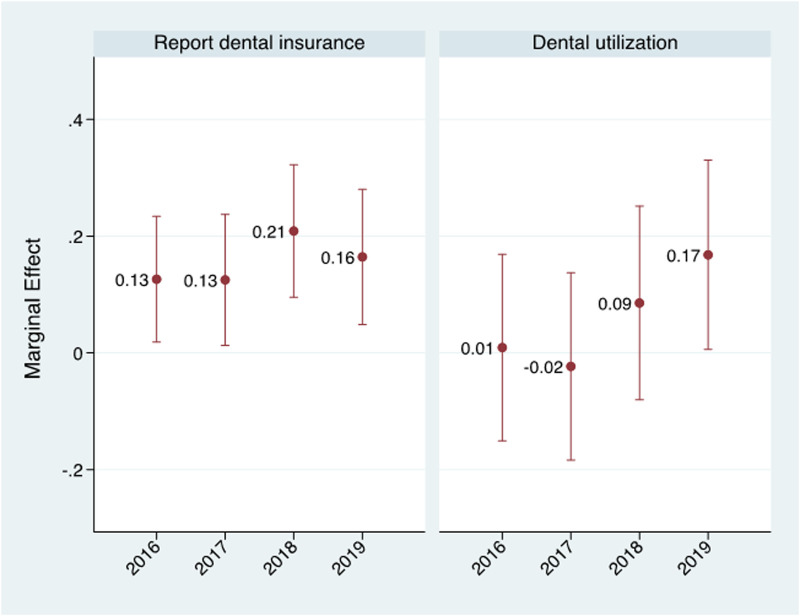
Average marginal effects for Medicaid × Year interaction variables from multivariable logistic regression estimating the impact of comprehensive dental benefit on report of dental insurance and dental care utilization during pregnancy among Medicaid-enrolled women. Note: Models for both outcomes controlled for age, race, ethnicity, education, location, marital status and year fixed effects.

Figure 3 presents the average marginal effects from the multivariable logistic regressions for both outcomes for Medicaid-enrolled women compared to women with private insurance. In each post-period year among Medicaid-enrolled women, the report of dental insurance was significantly higher compared to the pre-period than women with private insurance, with the largest difference of 21 percentage points in 2018 (marginal effect = 0.21, 95% CI = 0.10, 0.32). There was a 13 percentage point increase in 2016 and 2017 and a 16 percentage point increase in 2019 in the report of dental insurance among Medicaid enrolled women compared to the pre-period. Despite the immediate increase in reporting of dental insurance, there was no evidence of an effect on dental care utilization in the first two full years of the post-period. A nine percentage point increase was estimated for 2018, however, and the 17 percentage point estimated increase for 2019 was statistically significant. Both models were controlled for year fixed effects and sociodemographic variables. Full model results are available in Appendix Table 1.

A modification of the multiple logistic regression provides a formal test of the parallel trends assumption by including an additional interaction variable between Medicaid and calendar year 2014 and test the significance of this estimate; that is test whether there is a significant change in reporting dental insurance or dental care utilization before the advent of the comprehensive Medicaid dental benefit (20). For both the dental insurance (*t*-statistic = 0.24, *p* = 0.81) and dental care utilization (*t*-statistic = 0.86, *p* = 0.39) outcomes, we find no evidence of a significant change in outcomes for Medicaid enrollees relative to those with private insurance prior to the advent of the policy, thus providing support for the assumption of parallel trends.

## Discussion

In this study, we examined whether the comprehensive dental coverage benefit implemented in 2015 for Medicaid-enrolled pregnant women in Virginia was associated with an increase in dental insurance and dental care utilization during pregnancy using six years of state-level data. Findings show that self-reported dental insurance increased significantly for Medicaid-enrolled women in all post-period years, and dental care utilization saw 17 percentage points increase in 2019, 4 years after the dental benefit implementation. The gap in the report of dental insurance and dental care utilization in the post-period narrowed significantly between women with Medicaid and private health insurance.

Our stratified analyses findings clearly show that for both Medicaid enrollees and women with private health insurance, lack of dental insurance resulted in much lower dental care utilization. The results also pinpointed that even among women who reported dental insurance, dental care utilization among Medicaid enrollees remained lower than for women with private health insurance. These findings are suggestive of a different tendency for dental utilization among those who had access to insurance through Medicaid compared to those who obtained access outside Medicaid. Having dental coverage improves dental care use as seen from the findings in post-period years, but it does not fill the dental care utilization gap, suggesting additional barriers that impede dental care use during pregnancy among low-income women. These can include but are not limited to knowledge and attitudes towards oral health, awareness of dental coverage, confusion about available coverage, limited window of eligibility, and inability to find a dental provider who will take pregnant or Medicaid patients (21, 22). Our results highlight the need to address these additional barriers to further reduce dental care utilization disparities between Medicaid and privately insured women. Even though all Medicaid enrolled women had dental coverage in the post-period, even in the fourth year after the inception of the dental benefit, 25.6% of these women reported that they did not have coverage showing the extent of limited awareness among this population. These results concur with the recent study that found that 34% of reproductive-age women enrolled in Medicaid were unaware of available dental benefits in the Virginia Medicaid program after three years (13).

Moreover, the significant increase in reporting of the dental insurance was not accompanied by a commensurate increase in utilization. The impact of pregnancy dental benefit on dental care utilization was realized after four years. Possible explanations for this finding includes that, (1) providers' awareness, acceptance of Medicaid patients, and appointment availability may be limited in early years of dental benefit implementation, and (2) Medicaid-enrolled women's awareness of oral health importance and dental benefit grew over the years resulting in higher utilization than previous years. In addition to the provision of dental coverage, efforts should also include targeted outreach and education for patients and providers about the importance of oral health during pregnancy.

Previous studies have shown that expanding dental benefits in Medicaid positively affected dental utilization and reduced emergency visits among adults (23, 24). No study has examined pregnant women population and whether there was a time lag to observe the effect of policy on dental care utilization. This is the first study to use statewide PRAMS data to examine the impact of dental policy on dental care utilization among pregnant women and add the information on the time required to see policy effect.

Since July 1, 2021, Virginia Medicaid program covers comprehensive dental coverage for all qualifying adults. Examining utilization under this new policy may enhance understanding of the impact of dental coverage on utilization and oral health of Medicaid enrolled pregnant women as the 2021 dental benefit will provide coverage before, during, and after pregnancy and reduce barriers to continuity of care.

Although multiple years of data and robust quasi-experimental methods were used, the following study limitations should be noted. PRAMS is a self-reported survey and has associated biases of recall and reporting. There is a possibility that some women in the Medicaid group may have had dental coverage from other sources than Medicaid, but we do not expect it to differ during pre and post-periods. Since we only utilize data from one state, sample sizes for a few subgroups were small. However, we combined six years of data and used weighted estimates to account for small subgroups and improve estimate precision. It is important to note that even though data is from one state, the results apply to a broader context of the Medicaid population and can inform programs and policies in states planning to expand dental coverage for pregnant women and other adults. Further research should explore barriers to dental care utilization, examine dental care use for problem-based visits among pregnant women in different groups and identify ways to address them effectively.

This is the first study to use statewide PRAMS data to examine the impact of dental policy on dental care utilization among pregnant women. In conclusion, Virginia's Medicaid dental benefit implementation positively impacted awareness of a dental insurance benefit and increased dental care utilization among Medicaid-enrolled pregnant women. By adding dental benefit, Virginia eliminated a primary barrier to dental care utilization among Medicaid enrolled pregnant women, but additional work should be done in the Commonwealth of Virginia to increase awareness of the Medicaid dental benefit and address barriers to dental care utilization to promote oral health equity. The study results provide useful information for policymakers, clinicians, educators, and community partners to identify areas that need to be strengthened and inform the development of targeted initiatives to increase oral health utilization during pregnancy.

## Data Availability

The data analyzed in this study is subject to the following licenses/restrictions: PRAMS data can be requested directly through CDC or through the individual State health program. Requests to access these datasets should be directed to Virginia Department of Health at Epi-Comments@vdh.virginia.gov.

## Appendix

TABLE A1 Marginal effect estimates and 95% confidence interval for dental insurance and dental utilization outcomes from the multivariable logistic regression models. Virginia PRAMS 2013–2014 and 2016–2019 data.

**Table:**

Variables	Dental Insurance	Dental Utilization
Health Insurance		
Private	Ref.	Ref.
Medicaid	−0.34 (−0.44, −0.23)^c^	−0.12 (−0.24, −0.01)^a^
Medicaid × Time		
Medicaid × 2016	0.13 (0.02, 0.23)^a^	0.01 (−0.15, 0.17)
Medicaid × 2017	0.13 (0.01, 0.24)^a^	−0.02 (−0.18, 0.14)
Medicaid × 2018	0.21 (0.10, 0.32)^c^	0.09 (−0.08, 0.25)
Medicaid × 2019	0.16 (0.05, 0.28)^b^	0.17 (0.01, 0.33)^a^
Age		
21–25	Ref.	Ref.
26–30	0.02 (−0.04, 0.08)	0.05 (−0.03, 0.14)
31–35	0.04 (−0.02, 0.10)	0.07 (−0.01, 0.15)
36 and above	0.04 (−0.02, 0.11)	0.10 (0.01, 0.19)^a^
Race		
White	Ref.	Ref.
Black	0.03 (−0.02, 0.08)	−0.03 (−0.11, 0.04)
Asian/other	−0.04 (−0.10, 0.02)	−0.11 (−0.18, −0.04)^b^
Ethnicity		
Non-Hispanic	Ref.	Ref.
Hispanic	−0.07 (−0.14, −0.00)^a^	0.02 (−0.08, 0.12)
Education		
Bachelors or higher	Ref.	Ref.
Less than High School	−0.20 (−0.33, −0.07)^b^	−0.21 (−0.36, −0.05)^b^
High School Graduate	−0.03 (−0.09, 0.03)	−0.14 (−0.22, −0.07)^c^
Some College	−0.02 (−0.08, 0.03)	−0.15 (−0.22, −0.09)^c^
Location		
Urban	Ref.	Ref.
Rural	0.02 (−0.04, 0.09)	−0.04 (−0.12, 0.05)
Marital Status		
Married	Ref.	Ref.
Not Married	0.00 (−0.05, 0.05)	−0.06 (−0.13, 0.01)

Models for both outcomes are controlled for year-fixed effects.

^a^ Significant at the 0.05 level.

^b^ significant at the 0.01 level.

^c^ significant at the 0.001 level.
